# Risk of Premotor Symptoms in Patients with Newly Diagnosed PD: A Nationwide, Population-Based, Case-Control Study in Taiwan

**DOI:** 10.1371/journal.pone.0130282

**Published:** 2015-06-24

**Authors:** Yu-Hsuan Wu, Yi-Chu Liao, Yi-Huei Chen, Ming-Hong Chang, Ching-Heng Lin

**Affiliations:** 1 Section of Neurology, Taichung Veterans General Hospital, Taichung, Taiwan; 2 Department of Neurology, National Yang-Ming University School of Medicine, Taipei, Taiwan; 3 Department of Medical education and Research, Taichung Veterans General Hospital, Taichung, Taiwan; Biogen Idec, UNITED STATES

## Abstract

**Background:**

To evaluate the risk of premotor symptoms, namely rapid eye movement behavior disorder (RBD), constipation, and depression among patients with newly diagnosed Parkinson disease (PD).

**Methods:**

A total of 705 PD patients and 2,820 control subjects were selected from the Taiwan National Health Insurance Research Database. Patients were traced back for a maximum of 14 years to determine the diagnoses of RBD, depression, and constipation. Logistic regression analysis was used to identify risk of premotor symptoms for PD. Moreover, subgroup analyses were performed by dividing the patients into a middle-age onset group (≤ 64 years) and an old-age onset group (≥ 65 years). The associations between these premotor symptoms and age of PD onset were further examined.

**Results:**

An association was found between a history of premotor symptoms and newly diagnosed PD in which a high occurrence of premotor symptoms was identified in PD patients as compared to selected controls (4.3% vs. 1.2% for RBD, 40.4% vs. 24.0% for constipation, and 13.0% vs. 5.1% for depression). The strength of this association remained statistically significant after adjustment for potential confounders (3.69 fold risk for RBD, 2.36 for constipation, and 2.82 for depression, all p < 0.0001). The average interval between premotor symptoms and PD ranged from 4.5 to 6.2 years. RBD and depression carried higher risks for PD in the middle-age onset group than in the old-age onset group (7.20- vs. 2.24-fold risk for RBD, 6.06 vs. 1.40 for depression).

**Conclusion:**

The prevalence of premotor symptoms was higher among the PD patients than in the controls. Premotor symptoms appeared to be associated with a higher risk for PD in subjects with an earlier age of onset.

## Introduction

Parkinson disease (PD) is a neurodegenerative disorder characterized by a wide spectrum of motor symptoms [1]. However, certain non-motor symptoms might precede the development of motor symptoms by several years. These premotor symptoms could include rapid eye movement behavior disorder (RBD), depression, and constipation [2].

RBD is a type of parasomnia characterized by dream-enacting behaviors. The estimated prevalence of RBD is 15–30% for patients with PD during the early stage of the disease [3,4]. It has been reported that RBD as a premotor feature occurred in 38% of patients who developed PD after an average 5-year follow-up; on an extended follow-up, 81% of these patients finally developed neurodegenerative disorders [5]. Similarly, one retrospective study (n = 44) showed that 45% of the patients with RBD developed neurodegenerative disorders after a mean latency of 11.5 years from the initial symptoms of RBD, and PD constituted up to half of those diagnoses [6]. Depression and constipation are also commonly associated with the manifestations of PD. Several studies have shown that depressed patients carried a 2.2- to 3.2-fold increase in risk for developing PD as compared to non-depressed patients [7,8]. And according to a cohort study with a longitudinal follow-up of 24 years, the incidence of PD increased in subjects with decreasing bowel movement and frequent laxative use [9,10]. Whether premotor symptoms are early manifestations of PD or whether they should be considered as separate independent risk factors remains unclear. For this reason, our study had 3 aims: (1) to determine which of the premotor symptoms is the best predictor of future occurrence of PD, (2) to examine the interval between diagnosis of premotor symptoms and PD, and (3) to determine the associations between premotor symptoms and onset age of PD.

## Methods

### Study design

This was a nationwide, population-based, case-control study that used claims data.

### Data source

The National Health Insurance program was implemented in Taiwan in 1995 and it covered nearly 98% of the entire population of Taiwan in 2010 [11]. The National Health Insurance Research Database is a claim database of medical records, organized by the Bureau of National Health Insurance. This study used the Longitudinal Health Insurance Database 2005 which consists of all registration and original claims between 1996 and 2010 of a randomly selected group (n = 1 million) from the total registry in 2005 (n = 25.68 million). There were no statistical differences in age, sex, or average health care costs between the selected patients and the original enrollees by using the linear congruential random number generation function of the Sun Work Shop Compiler C 5.0. The distribution of study subjects were representative of the national population in Taiwan. The release of data was approved by the Ethics Committee of National Health Insurance, and the study protocol complied with the Declaration of Helsinki.

### Study population

Subjects with newly diagnosed PD from January 2006 to December 2010 were selected as the disease group. The medical records were traced back from 2010 to 1996. The diagnostic code of PD in the international classification of diseases, 9^th^ revision, clinical modification (ICD-9-CM) code was 332.0 [12]. The index date for the study group was indentified as the date of the first visit that resulted in PD diagnosis, between 2006 and 2010. To enhance diagnostic validity, only patients who received PD diagnosis in three or more consecutive visits at outpatient clinics or those diagnosed with PD during hospitalization were included as our PD subjects. We excluded subjects who took anti-dopaminergics more than three times within 3 months prior to the diagnosis of PD (Table A in S1 File). Subjects at risk for secondary or atypical Parkinsonism were also excluded (Table B in S1 File). Patients who had never received any anti-parkinsonian medications after the diagnoses of PD and those with incomplete data (sex, age) were also excluded.

Control subjects were the remaining patients during the same enrollment period. They were randomly selected to be age-, sex- and index-date-matched to the disease group and involved four times the number of subjects as compared to the patients with PD. The patients with a prior history of Parkinsonism, stroke, dementia, meningitis, encephalitis, head injury, hydrocephalus, toxic encephalopathy, brain tumor, and possible congenital disorders were not included. Patients who died before the index date or who had insufficient basic data were also not selected.

### Premotor symptoms

Patients in both groups were then individually traced back to check whether they had any diagnoses of depression (ICD-9-CM: 300.4, 309.0 occurring at least three or more times at outpatient clinics or more than once during hospitalization), constipation (ICD-9-CM: 564.0 and receiving prescriptions with laxatives at least three times) and RBD. The ICD-9 CM code for RBD is 327.42, however, it is not a familiar code for doctors in Taiwan. For avoiding this underestimation, we also included the subjects who received clonazepam every night at least three times, but in the absence of insomnia and anxiety (ICD-9-CM: 307.41–42, 780.52, 780.54, 3000, 29384, 30921). The codes for premotor symptoms were extracted in the years before diagnosis.

### Statistics

Stata version 11.1 was used to perform all statistical analyses. Student’s t test was used to compare the average age between cases and control groups. The Pearson chi-squared test was used to examine the distributions of sex, premotor symptoms (depression, constipation, RBD) and diabetes mellitus between patients with PD and control subjects.

The odds ratios (OR) of depression, constipation and RBD in PD were estimated by logistic regression analyses. Multivariate regressions adjusted for age, gender and diabetes mellitus were also conducted. All the three premotor symptoms were included in the same multivariate regression model to examine its impact on PD risks. The sensitivity and specificity of three premotor symptoms to predict PD were calculated.

Moreover, subgroup analyses were performed by dividing the patients into a middle-age onset group (≤ 64 years) and an old-age onset group (≥ 65 years) [13].

## Results

Table 1 shows the demographic characteristics and co-morbidities of the study participants. A total of 705 patients (343 men, 362 women) were newly diagnosed with PD among the randomly selected group (n = 882,086). The incidence rate per 100,000 person-years was 16.0, which is similar to that of a previous study (10.2 to 18 per 100,000 person-years) [14]. The average age at the time of PD diagnosis was 66 ± 15.1 years. Males accounted for 48.7% of the patients with PD. The average of prediagnostic phase in both groups was 12.51 years (the shortest one was 10 years; the longest one was 15 years). The control subjects (n = 2820) were randomly selected from the database to be age-, sex-, and index-date-matched to the patients with PD.

**Table 1:** Clinical characteristics of study subjects with and without PD (N = 3525).

Variable	Total (N = 3525) N (%)	Control subjects (N = 2820) N (%)	PD patients (N = 705) N (%)	p-value
**Age, years (mean±SD)**	66.0±15.3	66.0±15.4	66.0±15.1	0.93^a^
**Gender**				1.00
** Female**	1810 (51.3)	1448 (51.3)	362 (51.3)	
** Male**	1715 (48.7)	1372 (48.7)	343 (48.7)	
**Diabetes** ^b^	648 (18.4)	513 (18.2)	135 (19.1)	0.56

^a^ Student’s t test; chi-squared test for all other p-values.

^b^ Definition of diabetes: patients with diseases of the following ICD codes: 250/ A181.

PD: Parkinson’s disease, N: case number, %: percentage.

Results of the comparisons between patients with PD and control subjects in terms of depression, constipation, and RBD are summarized in Table 2. Among the 705 patients with PD, 30 had RBD, 92 had depression, and 285 had constipation. The occurrence of RBD in patients with PD (4.3%) was significantly higher than that in the non-PD group (1.2%) (Table 2, p < 0.0001). Similarly, the percent occurrences of constipation and depression were significantly higher in patients with PD than in control subjects (40.4% vs. 24.0% and 13.0% vs. 5.1%, respectively). The specificity for RBD to predict subsequent PD was as high as 98%, but the sensitivity was rather low (4.3%). Similarly, constipation and depression had high specificities (76% and 95%, respectively) and low sensitivities (40% and 13%, respectively) in the prediction of PD. After adjusting for age, sex, and comorbidity, the subjects with RBD carried a 3.69-fold risk for PD in comparison to those without RBD (Table 3, p < 0.0001). Subjects with constipation or depression were also at a significantly higher risk for PD with an odds ratio (OR) of 2.36 and 2.82, respectively, in comparison to those without depression or constipation (Table 3, p < 0.0001). When the three premotor symptoms (i.e., depression, constipation and RBD) were all included in the regression model, the ORs for RBD, constipation, and depression were 3.47, 2.19, and 2.48, respectively (Table 3, all p < 0.0001). This suggests that the three premotor symptoms were all significant and independent predictors of future PD risk.

**Table 2:** Comparisons of depression/constipation/RBD distribution between study subjects with and without PD.

Variable	Total(N = 3525) N (%)	Control subjects (N = 2820) N (%)	PD patients (N = 705) N (%)	p-value	Sensitivity (%)	Specificity (%)
**RBD**	63 (1.8)	33 (1.2)	30 (4.3)	**< .0001**	4.3	98
**Constipation**	963 (27.3)	678 (24.0)	285 (40.4)	**< .0001**	40	76
**Depression**	236 (6.7)	144 (5.1)	92 (13.0)	**< .0001**	13	95

Chi-squared test for p-values. RBD: Rapid eye movement behavior disorder, PD: Parkinson’s disease, N: case number, %: percentage.

**Table 3:** Odds ratio of depression/constipation/RBD associated with PD in logistic regression analysis.

	model 1 ^a^		model 2 ^b^	
Variable	Odds ratio	p-value	Odds ratio	p-value
**Age, years (mean±SD)**	1.00	0.13	1.00	0.13
**Gender**		0.96		0.96
** Female**	1.00		1.00	
** Male**	1.00		1.00	
**Diabetes** ^c^	1.00	0.97	1.00	0.97
**RBD**	3.69	**< .0001**	3.47	**< .0001**
**Constipation**	2.36	**< .0001**	2.19	**< .0001**
**Depression**	2.82	**< .0001**	2.48	**< .0001**

^a^ OR and p-values were adjusted for age, gender, diabetes.

^b^ OR and p-values were adjusted for age, gender, diabetes and depression, constipation.

^c^ Definition of diabetes: patients with diseases of the following ICD codes: 250/ A181.RBD: Rapid eye movement behavior disorder, PD: Parkinson’s disease.

The average time interval between the diagnosis of RBD and PD was 4.9 years (SD 4.0) (Table 4). For patients with constipation or depression, the average interval between premotor symptoms and the PD diagnosis were 6.2 years (SD 3.7) and 4.5 years (SD 3.5), respectively (Table 4). For those with RBD, 60.0% were diagnosed with PD at least 3 years after their initial diagnoses of RBD (Table 4). Among those with constipation or depression, 75.1% and 57.6%, respectively, had their premotor symptoms at least 3 years earlier than the diagnosis of PD.

**Table 4:** Time interval between the premotor symptoms and the PD diagnosis.

Variable	With Parkinson Disease		
Interval (years) (mean±SD)	Total N (%)	age≤64 N (%)	age≥65 N (%)
**RBD (N = 30)**	4.9±4.0	6.0±4.5	3.8±3.1
**≤1**	5 (16.7)	2 (13.3)	3 (20.0)
**1–3**	7 (23.3)	3 (20.0)	4 (26.7)
**>3**	18 (60.0)	10 (66.7)	8 (53.3)
**Constipation (N = 285)**	6.2±3.7	6.1±3.6	6.2±3.7
**≤1**	29 (10.2)	4 (6.4)	25 (11.3)
**1–3**	42 (14.7)	12(19.1)	30 (13.5)
**>3**	214 (75.1)	47(74.6)	167 (75.2)
**Depression (N = 92)**	4.5±3.5	4.8±3.4	4.2±3.7
**≤1**	17 (18.)	6 (12.2)	11 (25.6)
**1–3**	22 (23.9)	11 (22.5)	11 (25.6)
**>3**	53 (57.6)	32 (65.3)	21 (48.8)

RBD: Rapid eye movement behavior disorder, PD: Parkinson’s disease, N: case number, %: percentage.

In the middle-age onset group, subjects with RBD had a 7.20-fold risk for developing PD in the coming years (Table 5, p < 0.0001). However, in the old-age onset group, subjects with RBD carried a modest risk for PD (OR = 2.24, p = 0.02). Similarly, constipation was associated with a higher risk for PD in the middle-age onset group as compared to the old-age onset group (OR = 3.07 and 2.08, respectively; both p < 0.0001). Depression was significantly associated with future PD risk in the middle-age onset group (OR = 6.06, p < 0.0001), but not in the old-age onset group (OR = 1.40, p-value = 0.08). Therefore, premotor symptoms appear to carry an elevated risk for PD in subjects with an earlier age of onset.

**Table 5:** Comparisons and odds ratio of the depression/constipation/RBD distribution between study subjects with and without PD, category by age group.

	age≤64 (N = 1363)			age≥65 (N = 2162)		
Variable	Control subjects(N = 1114) /PD patiens(N = 249)	OR	p-value	Control subjects(N = 1706) /PD patiens(N = 456)	OR	p-value
	N (%)			N (%)		
**Age, years**	50.8±12.3/49.4±12.4	0.99	0.20	75.9±6.5/ 74.9±6.3	0.96	<0.0001
**Gender**						
**Female**	532 (47.8)/115(46.2)	1.00		916 (53.7)/247 (54.2)	1.00	
**Male**	582 (52.2)/134 (53.8)	1.17	0.30	790 (46.3)/208 (45.8)	0.96	0.68
**Diabetes** ^a^	120 (10.8)/26 (10.4)	0.84	0.52	393 (23.0)/109 (23.9)	0.99	0.95
**RBD**	8 (0.7)/15 (6.0)	7.20	**< .0001**	25 (1.5)/15 (3.3)	2.24	0.02
**Constipation**	99 (8.9)/63 (25.3)	3.07	**< .0001**	579 (33.9)/222 (48.7)	2.08	**< .0001**
**Depression**	40 (3.6)/49 (19.7)	6.06	**< .0001**	104 (6.1)/43 (9.4)	1.40	0.08

OR and p-values: logistic regression analysis. OR and p-values were adjusted for age, gender, diabetes and depression, constipation, RBD. RBD: Rapid eye movement behavior disorder, PD: Parkinson’s disease, N: case number, %: percentage.

^a^ Definition of diabetes: patients with diseases of the following ICD codes: 250/ A181.

## Discussion

There are three main findings of this study: 1) RBD, constipation, and depression were associated with higher risks for PD, and each risk factor independently predicted the occurrence of PD; 2) the average interval for premotor symptoms to develop PD were 4.5–6.2 years; and 3) RBD and depression carried higher risks for PD in the middle-age onset group as compared to the old-age onset group.

The strengths of the present study are its large sample size, long duration of observation, and accurate diagnosis. In addition, unlike previous studies which estimated the occurrence of premotor symptoms using questionnaires [15,16], the presence of premotor symptoms in this study was confirmed by diagnoses and prescriptions in the insurance claim database.

To our best knowledge, almost all previous reports for RBD were cohort study that described the conversion of RBD to PD. Only one recent case-control study reported that the prevalence of RBD in prediagnostic phase was less than one percent by extracting the codes for RBD. Due to smaller samples, RBD was therefore excluded from further examination [17]. In consideration of underestimation by extracting unfamiliar codes for RBD, the prescription for RBD was also included in our definition. Therefore, our finding of RBD prevalence before the diagnosis of PD was higher (4.3%).

Most previous studies found an approximately 2-fold increase in the risk of these premotor symptoms for developing PD. According to a study conducted in the Mayo Clinic, depressive disorder was present in 14.8% of patients with PD and accounted for a 1.9-fold increased risk for PD [15]. Savica et al. showed that 36.2% patients with PD had a history of constipation and were associated with an OR of 2.48 for PD [16]. Another study revealed about half (44.6%) of the cases of PD had constipation before the development of motor symptoms [18].

The variation in interval between the premotor symptoms and the diagnosis of PD seems to correlate with the nature of the initial symptoms. Most reports have shown an interval of 3.2 to 4.3 years between the diagnoses of RBD and PD [3.5], which is similar to the interval of 4.5 years found in the present study. Gaig and Tolosa showed that the symptoms of depression occur prior to PD diagnosis with an average gap of 3 to 6 years [19], which is in accordance with our findings of an interval of 4.5 years. However, Bugalho and Viana-Baptista found that patients with idiopathic RBD developed PD after a mean interval of 12 years from the date of the initial symptoms [3]. Furthermore, a long interval of 20 years preceding the diagnosis of PD has also been reported [19]. A previous study also showed a lag time of 7.9 years between the onset of constipation and the motor symptoms of PD [16].

It is intriguing to find that RBD and depression are associated with higher risks for PD in the middle-age onset group compared to the old-age onset group. This may be due to the high prevalence of RBD and depression in the general population of elderly (in the non-PD group, the occurrence among old-age vs. middle-age onset group was 1.5% vs. 0.7% for RBD, and 6.1% vs. 3.6% for depression; Table 5). As a consequence, the positive predictive value of RBD and depression were significantly lower in the old-age onset group than in the middle-age onset group (37.5% vs. 65.2% for RBD, 29.3% vs. 55.1%, for depression).

There is a strong correlation between non-motor symptoms and α-synuclein pathology, which lends support to a link between premotor symptoms and PD. Constipation is thought to be an early feature of PD and the deposits of misfolded α-synuclein in the enteric nervous system and dorsal motor nucleus of the vagus might precede the classic changes in midbrain and limbic areas [20]. A recent study described deposition of colonic α-synuclein from three old colonoscopy samples taken 2 to 5 years before diagnosis of clinical PD [21]. The dysfunction of the coeruleus/subcoeruleus complex and reticular nuclei may underlie the expression of depression and RBD in PD. The structure of the coeruleus/subcoeruleus complex has strong serotonergic input from caudal raphe nuclei, which is probably important for controlling moods, sleep-wake cycles, and behavioral responses to sensory stimuli [20].

There are nonetheless a number of limitations to our findings and report. First, our cross-sectional study design can only demonstrate associations between premotor symptoms and PD rather than a causal relationship. The possibility that premotor symptoms are only early manifestations of PD cannot be ruled out. Second, other potential factors contributing to PD, e.g., family history of PD, tobacco and coffee use, and environmental exposures, are not available in the National Health Insurance Research Database. Third, the National Health Insurance Research Database only allows us to trace back the premotor symptoms for a maximum of 14 years. Because premotor symptoms might precede PD by more than 15 years, the estimated interval in this study may not have been exact.

Whether the premotor symptoms are independent risk factors for PD or merely early manifestations of the prodromal phase in PD remains debatable. Our population-based, retrospective study found that the prevalence of RBD, constipation and depression were higher in the patients with PD than in the control subjects. RBD, constipation and depression are independent risk factors for PD, with RBD and depression carrying higher risks for PD in the middle-age onset group than in the old-age onset group. Therefore, clinical suspicion for PD and long-term follow-up are warranted in subjects with RBD, depression or constipation.

## Supporting Information

S1 File Drugs with high risk of extrapyramidal symptoms (Table A). Diseases with risk of secondary or atypical Parkinsonism(**Table B**).
(DOC)

## References

[1] Parkinson J. An essay on the shaking palsy. 1817. J Neuropsychiatr 2002 Spring;14(2):223–36. 10.1176/jnp.14.2.223 11983801

[2] Lang AE. A critical appraisal of the premotor symptoms of Parkinson’s disease: potential usefulness in early diagnosis and design of neuroprotective trials. Mov Disord 2011 4;26(5):775–83. 10.1002/mds.23609 21484865

[3] Iranzo A, Molinuevo JL, Santamaría J, Serradell M, Martí MJ, Valldeoriola F, et al Rapid-eye-movement sleep behaviour disorder as an early marker for a neurodegenerative disorder: a descriptive study. Lancet Neurol 2006 7;5(7):572–7. 16781987 10.1016/S1474-4422(06)70476-8

[4] Lavault S, Leu-Semenescu S, Tezenas du Montcel S, Cochen de Cock V, Vidailhet M, Arnulf I. Does clinical rapid eye movement behavior disorder predict worse outcomes in Parkinson’s disease? J Neurol 2010 7;257(7):1154–9. 10.1007/s00415-010-5482-y 20148335

[5] Schenck CH, Bundlie SR, Mahowald MH. Delayed emergence of a parkinsonian disorder or dementia in 81% of older men initially diagnosed with idiopathic rapid eye movement sleep behavior disorder: a 16-year update on a previously reported series. Sleep Med. 2013 8;14(8):744–8. 10.1016/j.sleep.2012.10.009 23347909

[6] Iranzo A, Molinuevo JL, Santamaría J, Serradell M, Martí MJ, Valldeoriola F, et al Rapid-eye-movement sleep behaviour disorder as an early marker for a neurodegenerative disorder: a descriptive study. Lancet Neurol 2006 7;5(7):572–7. 16781987 10.1016/S1474-4422(06)70476-8

[7] Nilsson FM, Kessing LV, Bolwig TG. Increased risk of developing Parkinson’s disease for patients with major affective disorder: a register study. Acta Psychi Scand 2001 11;104(5):380–6. 10.1034/j.1600-0447.2001.00372.x 11722320

[8] Leentjens AF, Van den Akker M, Metsemakers JF, Lousberg R, Verhey FR. Higher incidence of depression preceding the onset of Parkinson’s disease: a register study. Mov Disord 2003 4;18(4):414–8. 12671948 10.1002/mds.10387

[9] Abbott RD, Petrovitch H, White LR, Masaki KH, Tanner CM, Curb JD, et al Frequency of bowel movements and the future risk of Parkinson’s disease. Neurology 2001 8 14;57(3):456–62. 11502913 10.1212/wnl.57.3.456

[10] Abbott RD, Ross GW, White LR, Sanderson WT, Burchfiel CM, Kashon M, et al Environmental, life-style, and physical precursors of clinical Parkinson’s disease: recent findings from the Honolulu-Asia Aging Study. J Neurol 2003 10;250 Suppl 3:III30–9. 14579122 10.1007/s00415-003-1306-7

[11] Shen CC, Tsai SJ, Perng CL, Kuo BI, Yang AC. Risk of Parkinson disease after depression: A nationwide population-based study. Neurology 2013 10 22;81(17):1538–44. 10.1212/WNL.0b013e3182a956ad 24089392

[12] Guo Y-J, Liao Y-C, Lin C-H, Chang M-H. Initial Medication in Patients of Newly Diagnosed Parkinson’s Disease in Taiwan. PLoS ONE 2014 9 15;9(9):e107465 10.1371/journal.pone.0107465 25222829 PMC4164642

[13] Zhou MZ, Gan J, Wei YR, Ren XY, Chen W, Liu ZG. The association between non-motor symptoms in Parkinson’s disease and age at onset. Clin Neurol Neurosurg 2013 10;115(10):2103–7. 10.1016/j.clineuro.2013.07.027 23962754

[14] Van Den Eeden SK, Tanner CM, Bernstein AL, Fross RD, Leimpeter A, Bloch DA, et al Incidence of Parkinson's disease: variation by age, gender, and race/ethnicity. Am J Epidemiol 2003 6 1;157(11):1015–22. 12777365 10.1093/aje/kwg068

[15] Shiba M, Bower JH, Maraganore DM, McDonnell SK, Peterson BJ, Ahlskog JE, et al Anxiety disorders and depressive disorders preceding Parkinson’s disease: a case-control study. Mov Disord 2000 7;15(4):669–77. 10928577 10.1002/1531-8257(200007)15:4<669::aid-mds1011>3.0.co;2-5

[16] Savica R, Carlin JM, Grossardt BR, Bower JH, Ahlskog JE, Maraganore DM, et al Medical records documentation of constipation preceding Parkinson disease. A case-control study. Neurology 2009 11 24;73(21):1752–8. 10.1212/WNL.0b013e3181c34af5 19933976 PMC2788809

[17] Schrag A, Horsfall L, Walters K, Noyce A, Petersen I. Prediagnostic presentations of Parkinson's disease in primary care: a case-control study. Lancet Neurol. 2015 1;14(1):57–64. 10.1016/S1474-4422(14)70287-X 25435387

[18] Ueki A, Otsuka M. Life style risks of Parkinson’s disease: association between decreased water intake and constipation. J Neurol 2004 10;251 Suppl 7:vII18–23. 15505750 10.1007/s00415-004-1706-3

[19] Gaig C, Tolosa E. When does Parkinson’s disease begin? Mov Disord 2009;24 Suppl 2:S656–64. 10.1002/mds.22672 19877243

[20] Hawkes CH, Del Tredici K, Braak H. A timeline for Parkinson’s disease. Parkinsonism Relat Disord 2010 2;16(2):79–84. 10.1016/j.parkreldis.2009.08.007 19846332

[21] Shannon KM, Keshavarzian A, Dodiya HB, Jakate S, Kordower JH. Is alpha-synuclein in the colon a biomarker for premotor Parkinson's disease? Evidence from 3 cases. Mov Disord 2012 5;27(6):716–9 10.1002/mds.25020 22550057

