# Supplementary material for: Risk of Premotor Symptoms in Patients with Newly Diagnosed PD: A Nationwide, Population-Based, Case-Control Study in Taiwan
Source: PLoS One. 2015 Jun 24;10(6):e0130282. doi: 10.1371/journal.pone.0130282 (PMC4479561; doi:10.1371/journal.pone.0130282)
Supplement: S1 File — Diseases with risk of secondary or atypical Parkinsonism(Table B). (DOC) [file pone.0130282.s001.doc]

**Table S1. Drugs with high risk of extrapyramidal symptoms**

| Drug category | Generic drugs |
| --- | --- |
| Anti-emetics | Metoclopramide, Prochlorperazine |
| Neuroleptics | Haloperidol, Amisulpride, Flupentixol, Fluphenazine, Levomepromazine, Pimozide, Amisulpride, Thioridazine, Zuclopenthixol, Risperidone, Olanzapine, Aripiprazole |
| Calcium channel blocker | Flunarizine, Cinnarizine |
| Dopamine depleter | Reserpine, Tetrabenazine |
| Dopamine synthesis blocker | Methyldopa |

**Table S2. Diseases with risk of of 2^nd^** or atypical Parkinsonism

| Diseases | ICD-9-CM |
| --- | --- |
| Stroke | 430-438/A290-A294,A299 |
| Dementia | 290,331.0, 331.2/A210 |
| Meningitis, encephalities | 00321,﻿0065,﻿0130,﻿0131,﻿0132,﻿0133,﻿0136,﻿0360,﻿0361,﻿0460,﻿0461,﻿0462,﻿0463,﻿047,﻿0490,﻿0491,0520,﻿0530,﻿0543,﻿05472,﻿0550,﻿05601,﻿062,﻿063,﻿064,﻿0721,﻿0722,﻿09041,﻿09042,﻿0941,﻿0942,﻿09481,﻿09482,﻿09487,﻿09882,﻿10081,﻿11283,﻿1142,﻿11501,﻿11511,﻿11591,﻿1300,﻿1390,﻿320,321,322,323,3240,3249,﻿325,326 |
| Head injury | 800, 801, 803, 804, 850, 851, 852, 853, 854 |
| Hydrocephalus | 742.3, 741.0, 331.3, 331.4 |
| Brain tumor | 191, 192.0, 192.1, 192.8, 192.9, 194.3, 194.4, 198.3, 237.0, 237.1, 237.5, 237.6, 237.9, 239.6, 239.7, 2250, 2252, ﻿2273, 2274, ﻿22802 |
| Congenital or hereditary disorders | 2750, 2751,﻿ 3334,﻿ 334, ﻿740 |
| Hypoxic encephalopathy | 348.1, 997.01, 639.8, 669.4, 768.7, 779.2 |
